# Enhanced Piezoelectric Effect in P(VDF-TrFE) through Synergistic Templating by PEDOT:PSS and Paper

**DOI:** 10.1021/acsaelm.6c00088

**Published:** 2026-03-30

**Authors:** Xiangyi Wu, M. D. Hashan C. Peiris, Pravini S. Fernando, Anju Sharma, Joab Dorsainvil, Ahyeon Koh, Manuel Smeu, Jeffrey M. Mativetsky

**Affiliations:** † Materials Science and Engineering, 14787 Binghamton University, Binghamton, New York 13902, United States; ‡ Department of Physics, Applied Physics and Astronomy, Binghamton University, Binghamton, New York 13902, United States; § Department of Biomedical Engineering, Binghamton University, Binghamton, New York 13902, United States; ∥ Small Scale Systems Integration and Packaging Center, Binghamton University, Binghamton, New York 13902, United States

**Keywords:** P(VDF-TrFE), PEDOT:PSS, piezoelectric paper composite, templated growth, self-poling

## Abstract

To reduce the harm of electronic waste, there is impetus to expand the use of biodegradable substrates, biocompatible materials, and battery-free devices. In this study, a piezoelectric paper composite is produced by integrating polyvinylidene fluoride-trifluoroethylene [P­(VDF-TrFE)] with the conducting polymer poly­(3,4-ethylenedioxythiophene) polystyrenesulfonate (PEDOT:PSS) and wood fiber paper. It is shown that the combined effect of PEDOT:PSS and paper leads to an enhanced out-of-plane piezoelectric coefficient in P­(VDF-TrFE), without the need for electric poling. The enhanced piezoelectric performance arises from a synergistic templating effect of PEDOT:PSS and paper that induces a sharp increase in the population of edge-on β phase P­(VDF-TrFE) crystallites. The templating effect relies on supramolecular interactions between PSS and paper that lead to PEDOT enrichment and a linear PEDOT conformation at the interface with P­(VDF-TrFE). Computational modeling reveals that linear PEDOT promotes β phase PVDF growth over the α phase due to a greater availability of hydrogen bonds. The synergistic templating of P­(VDF-TrFE) on PEDOT:PSS-coated paper opens avenues for tuning the properties of piezoelectric paper.

## Graphical abstract

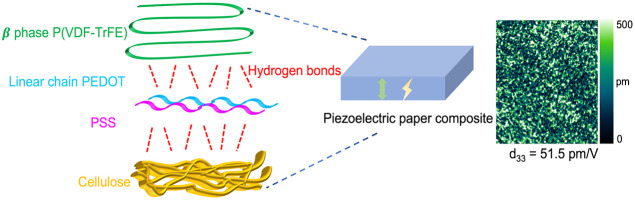

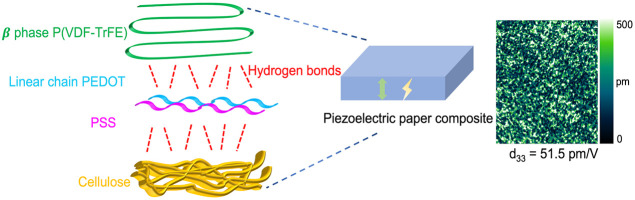

## Introduction

As the pervasiveness of electronics continues to increase, so does the amount of electronic waste generated.^1,2^ In response to this growing environmental concern, the inclusion of biodegradable and biocompatible materials as well as battery-free power sources have emerged as promising solutions.^3,4^ These approaches are especially relevant for devices with short life cycles, such as single-use medical sensors, or devices with low power requirements, such as Internet of things technologies.

Paper is a promising substrate for reducing the ecological footprint of electronics because it is biodegradable, recyclable, produced using a renewable source, wood pulp, and it is resistant to solvents, making it compatible with low-energy print processes.^5,6^ Further benefits of paper include its light weight, mechanical flexibility, and low cost. Paper primarily consists of cellulose, a linear polysaccharide, that forms microfibers through hydrogen bonding. To date, the field of paper electronics mainly treats paper as a conventional substrate, on which device layers can be deposited and patterned to produce wearable sensors,^7^ transistors,^8^ and displays.^9^ A less common, but promising approach is to instead use paper as a scaffold that can be infiltrated with functional materials, to create functional paper composites.^10,11^

The hydroxyl network found in paper can also be used to anchor functional organic coatings. For instance, carboxylic acid-functionalized carbon nanotubes have been attached to paper through hydrogen bonding, to produce humidity sensors.^12^ Furthermore, hydrogen bonding between poly­(vinylidene fluoride) (PVDF) and cellulose was noted to increase the thermal stability of composite films used in triboelectric nanogenerators.^13^ In addition to hydrogen bonding, π–π stacking, ionic interactions, entropic contributions, and environmental conditions can also play a role in mediating interactions between cellulose and organic coatings.^14^ There is untapped potential to employ these interfacial interactions to guide molecular assembly, promote intermolecular order, and enhance the performance of functional coatings on paper.

The integration of piezoelectric polymers with paper can combine the benefits of both materials. Piezoelectric materials that convert mechanical energy to electric energy offer an alternative to batteries in applications where wasted mechanical forces or vibrations can be put to use.^15^ Semicrystalline piezoelectric polymers, such as PVDF and its copolymers, have been studied extensively for energy harvesting and self-powered sensing due to their mechanical flexibility, biocompatibility, and ease-of-processing.^16^ Among the five crystal polymorphs of PVDF,^17^ the β phase bears an all-*trans* chain configuration with CF_2_ groups on one side of the chain and CH_2_ groups on the other, creating a high dipole moment and a pronounced piezoelectric effect.^18^ Unlike PVDF, the copolymer polyvinylidene fluoride-trifluoroethylene [P­(VDF-TrFE)] thermodynamically favors the ferroelectric β phase,^18^ since the TrFE units induce steric hindrance that impedes the formation of the nonpolar α phase.^19,20^ Dipole alignment is typically induced by poling with a large electric field (50 MV/m or greater), e.g., during electrospinning^15^ or after film deposition.^21^ It has been shown that the addition of cellulose nanocrystals can improve the piezoelectricity of electrospun PVDF by promoting β phase crystallization.^22,23^ Wet spun PVDF/cellulose composites have also been observed to exhibit a piezoelectric response without electrical poling.^24^ So far, however, studies have not examined how intermediate coatings can mediate the interactions between piezoelectric polymers and paper to enhance piezoelectric performance.

In this work, we employ a biocompatible conducting polymer,^25^ poly­(3,4-ethylenedioxythiophene) polystyrenesulfonate (PEDOT:PSS), together with wood fiber paper to template the growth of P­(VDF-TrFE). The combined influence of PEDOT:PSS and paper leads to an increase in P­(VDF-TrFE) β phase content, enhanced crystalline order, and a piezoelectric coefficient of up to 51.5 pm/V, without electrical poling. These advantages are absent or much reduced when PEDOT:PSS or paper are used separately. The synergistic templating of P­(VDF-TrFE) by PEDOT:PSS and paper is attributed to an abundance of linear chain PEDOT at the PEDOT:PSS/P­(VDF-TrFE) interface that is induced by the underlying paper. First-principles modeling combined with molecular dynamics shows that β phase PVDF growth on linear PEDOT is favored over the α phase because of a greater availability of hydrogen bonds. The demonstrated multistage templated growth offers a new means of tuning the growth and properties of piezoelectric polymers.

## Experimental Methods

### Sample Preparation

The preparation of PEDOT:PSS-saturated paper followed a previously outlined procedure.^11^ Briefly, dimethyl sulfoxide (DMSO, purity ≥99.9%) from EMD Millipore Corporation and Zonyl FS-300 fluorinated surfactant were added to PEDOT:PSS solution (Heraeus Clevios P VP AI 4083) with a DMSO/surfactant/PEDOT:PSS volume ratio of 5:1:100. A 0.45 μm syringe filter was used to remove aggregates and then the solution was stirred at 300 rpm for 15 min before deposition onto virgin wood fiber paper (Kimwipe, Kimtech Science) and drying under ambient conditions. P­(VDF-TrFE) powder with 25 mol % TrFE (solvene 250/400) purchased from Sigma-Aldrich was dissolved in dimethylformamide and acetone with a solvent volume ratio of 3:2 and solution concentration of 62 mg/mL. The P­(VDF-TrFE) solution was then stirred at 300 rpm overnight at room temperature. P­(VDF-TrFE) was dip-coated onto PEDOT:PSS-saturated paper, while fixed to a glass slide, by using a motorized control stage at room temperature, with a fixed withdrawal speed of 3.2 cm/min unless otherwise noted. Prior to withdrawal, the paper-based samples were first submerged in the P­(VDF-TrFE) solution for 15 s to ensure uniform wetting of the solution throughout the porous fiber network. After the dip coating, the samples were dried horizontally under ambient conditions for 3 h. The P­(VDF-TrFE)-coated PEDOT:PSS/paper was then annealed at 130 °C for 2 h to promote the crystallization of β phase P­(VDF-TrFE).^26^ Glass slides, from Fisher Scientific, and glass coated with indium tin oxide (ITO), from Colorado Concept Coating LLC, were cleaned in acetone, isopropanol, and water by sonication for 5 min in each solvent, dried under nitrogen gas flow, and then treated with UV-ozone for 15 min. For samples on glass and ITO/glass, the PEDOT:PSS solution was drop-casted onto the substrates and dried under ambient conditions overnight. Subsequently, P­(VDF-TrFE) was dip-coated onto PEDOT:PSS-coated glass or ITO/glass and annealed under the same conditions as the paper-based samples.

### Characterization

Atomic force microscopy (AFM) and piezoresponse force microscopy (PFM) were implemented under ambient conditions using a CombiScope AFM from AIST-NT with Pt-coated 0.2 N/m cantilevers (Budget Sensors ContE-g). PFM was carried out with an AC bias of 5 V and a normal force of 7 nN. PFM was conducted at three different locations for each sample, with each measurement comprising 200 × 200 data points. Force–distance curves were recorded at 100 sample positions and the average slope was used to calibrate the cantilever displacement and quantify the piezoelectric coefficient (*d*_33_) for each sample. Scanning electron microscopy (SEM) and energy-dispersive X-ray spectroscopy (EDS) were performed using a Zeiss Super-55VP SEM with a beam voltage of 15 kV. To prevent surface charging during SEM observation, carbon was coated onto the samples. Raman spectroscopy was performed using a Renishaw inVia confocal Raman microscope with a 785 nm laser focused to a 1.2 μm spot, an integration time of 5 s, and a laser intensity of 8.36 × 10^8^ W m^–2^. Attenuated total reflection Fourier-transform infrared spectroscopy (ATR-FTIR) measurements employed an alpha II platinum ATR-FTIR spectrometer. X-ray photoelectron spectroscopy (XPS) was implemented with a PHI 5000 VersaProbe under ultrahigh vacuum (10^–7^ Pa) with a 50 W monochromatic Al Kα (1486.6 eV) X-ray source. XPS spectra were calibrated to the sp^2^ carbon peak (284.8 eV). Grazing incidence wide-angle X-ray scattering (GIWAXS) was performed at the 11-BM beamline of the National Synchrotron Light Source II (NSLS-II) at Brookhaven National Laboratory. An X-ray wavelength of 0.09184 nm was used with an incident angle of 0.1°. The distance between sample and detector was 257 mm and the exposure time was 10 s. The scattering profiles were obtained using Fit2d GIWAXS software.^27^

### Computation

The interactions between PVDF molecular chains and PEDOT:PSS were modeled using the Vienna *Ab Initio* Simulation package (VASP).^28,29^ Projector augmented wave (PAW) potentials were used to simulate the ionic cores^28,30^ and the Perdew–Burke–Ernzerhof (PBE) generalized gradient approximation (GGA) provided the exchange and correlation functional^31,32^ with a Γ-centered 1 × 1 × 1 Monkhorst–Pack *k*-point grid used for all structural relaxations and energy calculations.^33,34^ The Brillouin zone was sampled using Gaussian smearing.

All density functional theory (DFT) calculations were performed using a plane wave energy cutoff set at 450 eV, with convergence testing for the system for the *k*-point grid, and the plane wave energy cutoff was set at 1 meV/atom.Van der Waals interactions were included via the Grimme D3 approach (PBE-D3) with Becke-Johnson damping in all calculations.^35^ The initial periodic structures for the α and β phases were obtained from our past work with full structural relaxations that matched the current experimental and computational literature (see Supporting Information Figure S11 for radial distribution function (RDF) plots).^36–38^

Given the scarcity of computed PEDOT:PSS composite structures in the literature, we ran short *ab initio* molecular dynamics (AIMD) trajectories of the PEDOT:PSS composite with several initial configurations (by varying the initial positions of the HSO_3_^–^ functional groups in the main chain as well as the ortho-para positioning, see Figure S12) for ∼5 ps for equilibration. Thereafter, a full structural relaxation of the final snapshot of the AIMD trajectory was performed, and the model that was both energetically stable and consistent with the linear lattice distances of the α and β PVDF chains, was used for subsequent calculations.

The α and β PVDF chains and the PEDOT:PSS single-molecule chains were first relaxed individually, and their per-repeat unit distance was calculated. Thereafter, the per repeat unit distances were calculated for each molecule. Based on these values, the appropriate number of repeat units were used in each system (α PVDF with PEDOT:PSS and β PVDF with PEDOT:PSS). This was done to ensure that the lattice vector mismatch for the PVDF and PEDOT:PSS chains were kept to a minimum. See Table S1 for details. In each of these systems, a vacuum region above 12 Å in each direction was maintained to prevent interaction between the periodic images.

All of the AIMD calculations were performed with a plane wave energy cutoff set at 450 eV using a Γ-centered *k*-point. Each system considered under AIMD was simulated for a minimum duration of 18 ps, utilizing a time step of 1 fs. Bond distances were calculated using a Python script developed in-house. The results were initially cross-checked with the VESTA and Ovito visualization software to ascertain accuracy.^39,40^

## Results and Discussion

In this study, we employ the conducting polymer complex PEDOT:PSS and wood fiber paper to template the growth of P­(VDF-TrFE). The molecular structure of PEDOT:PSS and P­(VDF-TrFE) are shown in Figure 1a. PEDOT:PSS is held together by ionic bonds between positively charged thiophene rings along the PEDOT chain and negatively charged sulfonyl groups along PSS. In the P­(VDF-TrFE) copolymer, the TrFE units distribute randomly along the PVDF chains. The prepared P­(VDF-TrFE)/PEDOT:PSS/paper, which we refer to as templated P­(VDF-TrFE), was light gray as shown in Figure 1b. A cross-sectional SEM view showing the microstructure of templated P­(VDF-TrFE) can be seen in Figure 1c. A fibrous and lamellar structure can be seen, and the thickness of the paper is about 74 μm. The corresponding EDS fluorine map (Figure 1d) shows that P­(VDF-TrFE) is mainly concentrated within the first 25 μm of the paper but does penetrate through the entire thickness of the paper. The EDS sulfur map (Figure 1e) shows that PEDOT:PSS uniformly coats the paper throughout its entire depth.

**Figure 1:**
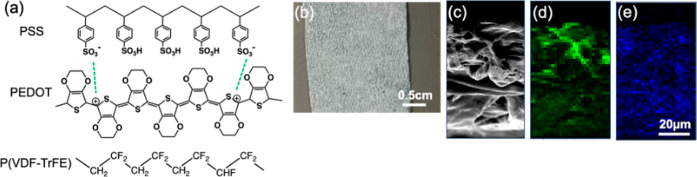
(a) Molecular structure of PEDOT:PSS and P­(VDF-TrFE). (b) Photograph, (c) cross-sectional SEM, (d) EDS fluorine map, and (e) EDS sulfur map of templated P­(VDF-TrFE).

For insight into the morphology and piezoelectric performance of templated P­(VDF-TrFE), we simultaneously measured the AFM topography and PFM response for P­(VDF-TrFE) coated onto PEDOT:PSS/paper (Figure 2a,d), paper (Figure 2b,e), and ITO (Figure 2c,f). In each case, the P­(VDF-TrFE) forms short needle-like structures, which is consistent with previous reports of P­(VDF-TrFE) with β phase content.^41,42^ We see that the P­(VDF-TrFE) on PEDOT:PSS/paper exhibits the greatest PFM response, followed by P­(VDF-TrFE) on paper and P­(VDF-TrFE) on ITO. The brightest features in Figure 2d, which correspond to the most piezoelectrically active regions, are attributed to β phase P­(VDF-TrFE) crystallites bearing an edge-on molecular orientation that favors an out-of-plane piezoelectric response. As shown in the Supporting Information (Figures S3 and S4), a higher P­(VDF-TrFE) concentration or dip-coating withdrawal rate produced thicker, rougher coatings, whereas a dilute solution or slower withdrawal rate yielded smoother but thinner layers; in each case, the piezoelectric response was reduced relative to the optimized condition.

**Figure 2:**
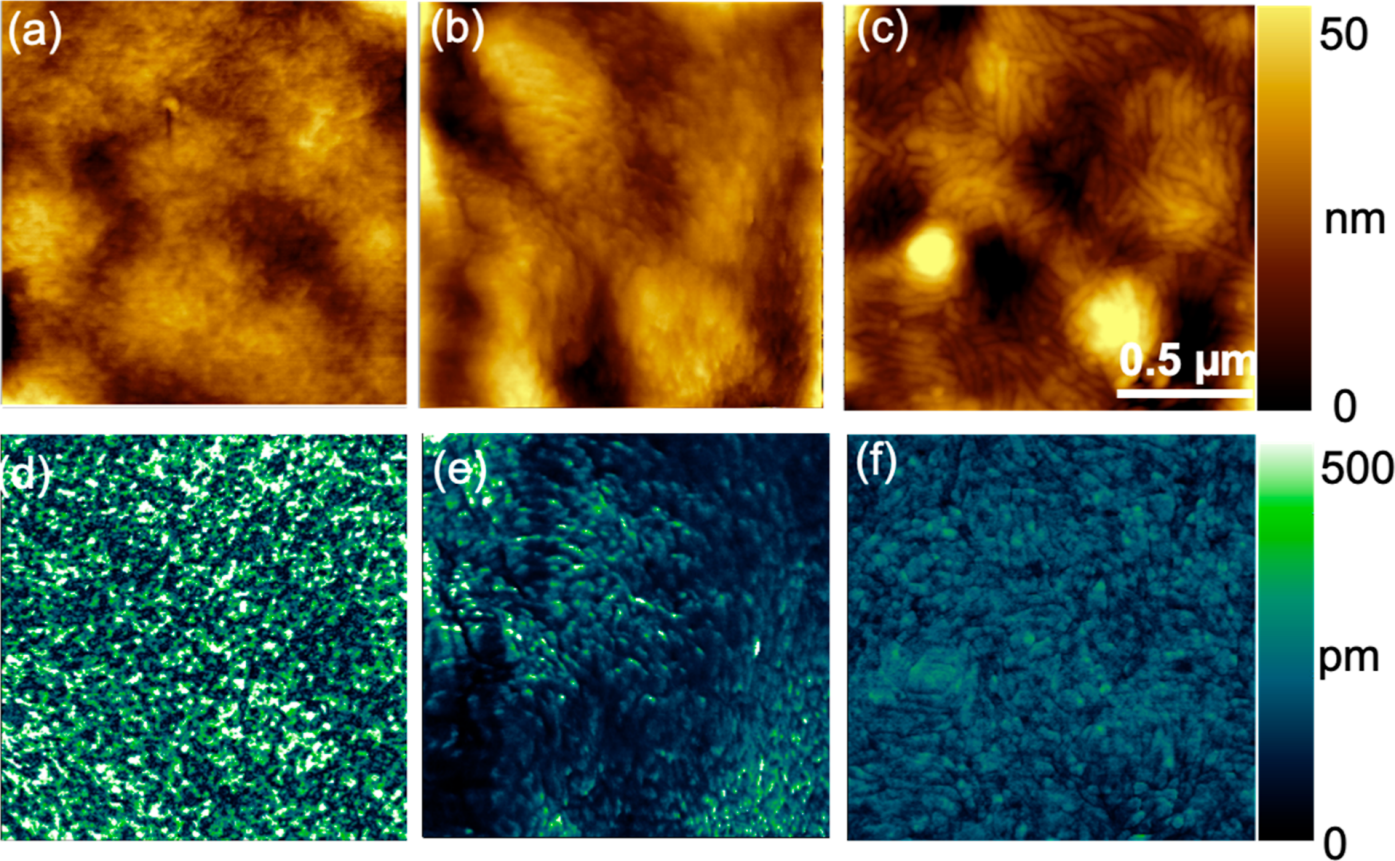
(a–c) AFM topography and (d–f) PFM map of P­(VDF-TrFE) on PEDOT:PSS/paper (left), paper (middle), and ITO (right).

The average piezoelectric coefficient *d*_33_ was determined by dividing the average PFM amplitude by the driving voltage. The *d*_33_ for P­(VDF-TrFE) coated onto PEDOT:PSS/paper, paper, and ITO is 51.5 ± 3.6 pm/V, 39.1 ± 4.0 pm/V, and 31.6 ± 0.6 pm/V, respectively, showing that paper has a positive effect on the piezoelectric performance of P­(VDF-TrFE), while PEDOT:PSS/paper exhibits the greatest effect. Interestingly, as shown in the Supporting Information (Figure S1), the *d*_33_ for P­(VDF-TrFE) on PEDOT:PSS-coated ITO is 33.2 ± 0.7 pm/V, similar to the case for P­(VDF-TrFE) on ITO. These results indicate that PEDOT:PSS only enhances the piezoelectric performance of P­(VDF-TrFE) when combined with paper to produce a synergistic effect. It should also be noted that paper itself is piezoelectric.^43^ For these samples, however, the piezoelectric response of paper represents only a small portion of the overall response, with the average *d*_33_ of 5.5 ± 1.1 pm/V for pristine paper (see Supporting Information Figure S2).

When PEDOT:PSS/paper templating is used, the piezoelectric performance falls short of the case for state-of-the-art interface engineered P­(VDF-TrFE)/PEDOT:PSS composites with electrical poling, which can attain a *d*_33_ as high as 86 pm/V.^26^ Nevertheless, our system reaches 60% of this value without any poling applied. For comparison, PVDF composites with cellulose nanocrystals and no poling attained a *d*_33_ of 26.2 pm/V.^24^

To elucidate the origin of the enhanced piezoelectric performance in templated P­(VDF-TrFE), we first performed ATR-FTIR measurements to examine the electroactive phase content. Figure 3 shows the ATR-FTIR spectra for P­(VDF-TrFE) on PEDOT:PSS/paper and P­(VDF-TrFE) on paper, with scaled substrate spectra subtracted; the substrate spectra are shown in Figure S5. The characteristic absorption band at 766 cm^–1^ is attributed to the α phase of P­(VDF-TrFE),^44^ while the band at 840 corresponds to the electroactive phases, β and γ. The bands at 1232, and 1273 cm^–1^ are associated with the γ and β phase, respectively. All three phases contribute to the 880, 1183 and 1400 cm^–1^ bands.^44–46^ On PEDOT:PSS/paper, the intensity of all identified P­(VDF-TrFE) bands increases, while the electroactive phase band at 840 cm^–1^ increases by a greater amount than the α phase band at 766 cm^–1^.

**Figure 3:**
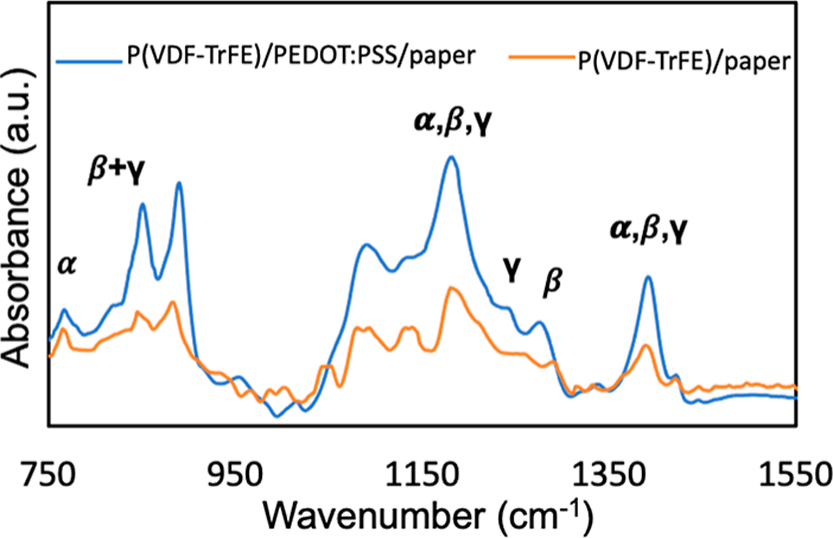
ATR-FTIR spectra for P­(VDF-TrFE) on PEDOT:PSS/paper and P­(VDF-TrFE) on paper with scaled substrate spectra subtracted.

To quantify the electroactive phase content F­(EA), we used the Lambert–Beer equation

1 $$F\left(EA\right)=\frac{A_{840}}{\left(\frac{K_{840}}{K_{766}}\right)A_{766}+A_{840}}$$

where *A*_766_ and *A*_840_ represent the absorbance intensities at 776 and 840 cm^–1^, respectively. *K*_766_ = 6.1 × 10^4^ cm^2^ mol^–1^ and *K*_840_ = 7.7 × 10^4^ cm^2^ mol^–1^ are the absorption coefficients at the corresponding wavenumbers.^44^ Using this approach, the electroactive phase content for P­(VDF-TrFE) on paper is 45.6%, while the electroactive phase content for P­(VDF-TrFE) on PEDOT:PSS/paper is 62.5%. For comparison, the electroactive phase content of P­(VDF-TrFE) on glass and on PEDOT:PSS/glass are 41.3% and 45.2%, respectively (see Supporting Information Figure S6).

Quantification of the β and γ phase content can be performed by calculating the peak-to-valley height ratio between the bands at 1273 and 1232 cm^–1^ and their nearest valleys, as demonstrated in eqs 2 and 3

2 $$F\left(\beta \right)=F\left(EA\right)\times \frac{△H_{\beta }}{△H_{\beta }+△H_{\gamma }}\times 100\%$$

3 $$F\left(\gamma \right)=F\left(EA\right)\times \frac{△H_{\gamma }}{△H_{\beta }+△H_{\gamma }}\times 100\%$$

where, Δ*H*_β_ and Δ*H*_γ_ are the intensity differences between the band at 1273 cm^–1^ and the nearest valley at 1260 cm^–1,^ and the band at 1232 cm^–1^ and the nearest valley at 1223 cm^–1^, respectively.^47^ Based on this calculation, the β and γ phase content is 41.0% and 4.6% on paper, and 50.0% and 12.5% on PEDOT:PSS/paper.

The greater β phase content in the sample containing PEDOT:PSS and paper is consistent with the PFM results that show superior piezoelectric performance in samples containing both PEDOT:PSS and paper, and less enhancement when PEDOT:PSS or paper are used separately. A recent study of P­(VDF-TrFE)/PEDOT:PSS blends showed that the electroactive (β and γ) phase content decreases with the addition of PEDOT:PSS.^48^ However, in our case, the combined effect of PEDOT:PSS and paper increases the electroactive phase content of P­(VDF-TrFE).

GIWAXS was carried out to determine the effect of the substrate on P­(VDF-TrFE) crystallization. GIWAXS patterns for P­(VDF-TrFE) on paper and on PEDOT:PSS/paper are shown in Figure 4a,b, respectively. Figure 4c shows the corresponding intensity profiles. The ring centered at *q* = 1.22 Å^–1^ corresponds to the α phase (100) reflection, while the ring at *q* = 1.44 Å^–1^ corresponds to overlapping β phase (200)/(110) and weaker γ phase (110) reflections.^47,49^ The faint rings at *q* = 2.34 and 2.74 Å^–1^ correspond to α phase (130) and γ phase (004) reflections, respectively. These reflections are much stronger for P­(VDF-TrFE) on PEDOT:PSS/paper than on paper, showing that the PEDOT:PSS/paper substrate promotes P­(VDF-TrFE) crystallization. For comparison, GIWAXS patterns for P­(VDF-TrFE) on PEDOT:PSS/glass and glass (Supporting Information Figure S7) exhibit weak α phase reflections and no visible β phase reflections, indicating that PEDOT:PSS/paper and paper help induce the formation of β phase crystallites. In addition to the primary peaks, there is an amorphous halo in the *q* = 1–1.5 Å^–1^ range. To determine the β phase P­(VDF-TrFE) crystallite size on PEDOT:PSS/paper and paper we used the Scherrer equation with the radial full width at half-maximum of the β phase (200)/(110) peak along *q*_*x*_ = 0. This analysis reveals a β phase crystallite size of 21.6 nm on PEDOT:PSS/paper, which is notably larger than that on paper (13.6 nm).

**Figure 4:**
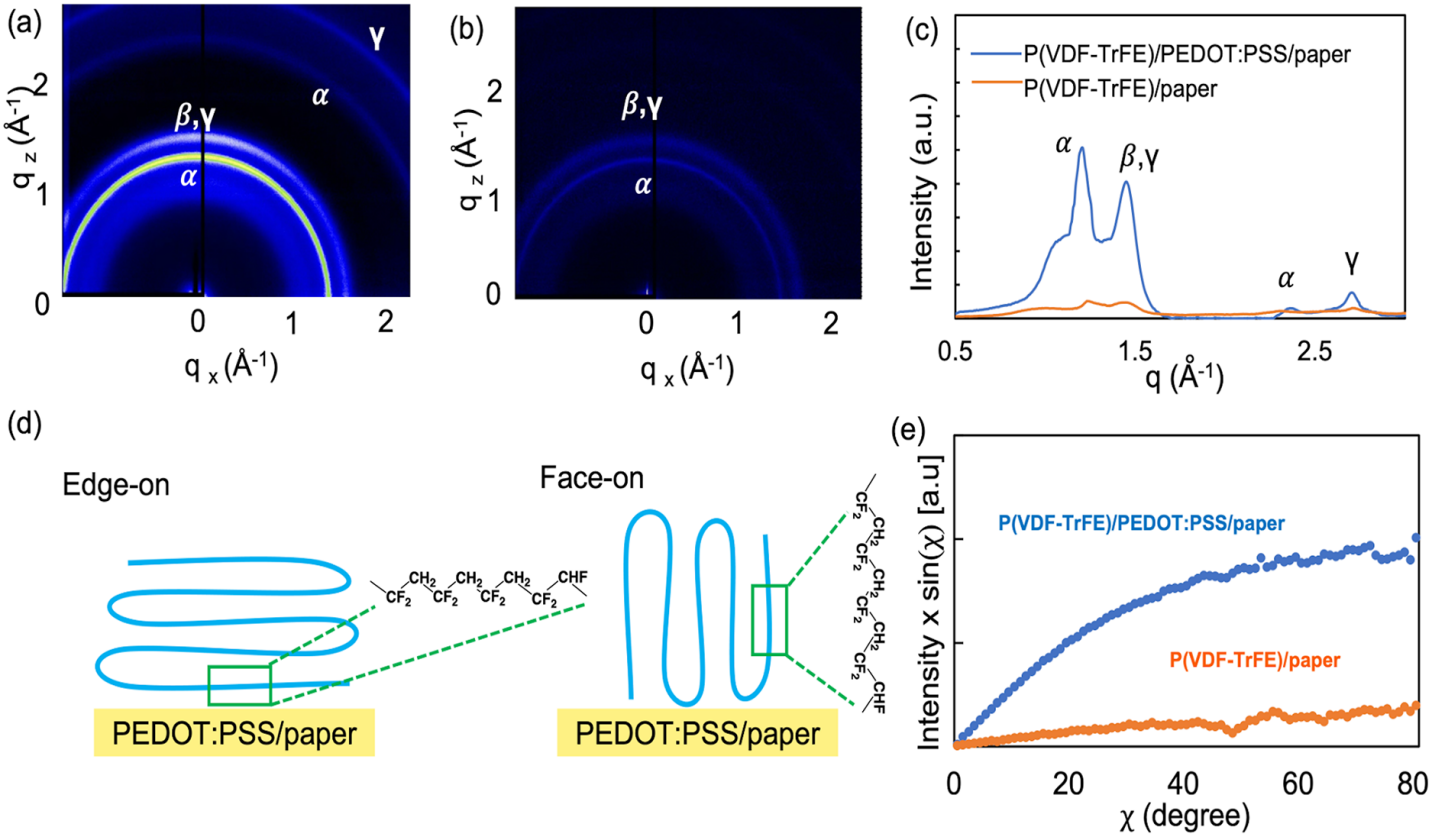
GIWAXS patterns for (a) P­(VDF-TrFE) on PEDOT:PSS/paper and (b) P­(VDF-TrFE) on paper. (c) Integrated intensity of (a,b) as a function of *q*. (d) Schematic depicting edge-on and face-on P­(VDF-TrFE) on PEDOT:PSS/paper. (e) Azimuthal integration of the P­(VDF-TrFE) (200) peak along χ, with the correction factor sin­(χ).

The orientation of the β phase plays a key role in dictating the piezoelectric properties of P­(VDF-TrFE). When P­(VDF-TrFE) lamellae adopt an edge-on orientation (Figure 4d), with the polymer chains preferentially oriented parallel to the substrate, an out-of-plane dipole is produced, promoting out-of-plane piezoelectric activity.^50^ The diffraction rings in Figure 4a,b indicate that the P­(VDF-TrFE) on paper and PEDOT:PSS/paper adopts a distribution of molecular orientations. To quantify the edge-on crystallite population, we analyzed the (200)/(110) β phase peak of the (*q*_r_, *q*_*z*_) GIWAXS patterns (Supporting Information Figure S8) as a function of azimuthal angle (χ), which was adjusted by multiplying the integrated intensity by sin­(χ),^52,53^ as shown in Figure 4c. The sin­(χ) term ensures that the entire reciprocal space is taken into account.^51,52^ Intensities at χ smaller than 45° are integrated to obtain the edge-on population and intensities at χ larger than 45° are integrated to obtain the face-on population. With this approach, we find that the edge-on crystallite population increases by a factor of 5.8, from 630 au on paper to 3660 au on PEDOT:PSS/paper, which is consistent with the improved out-of-plane piezoelectric coefficient for templated P­(VDF-TrFE).

To examine the role of interfaces in increasing the piezoelectric coefficient, β phase content, and crystallinity of templated P­(VDF-TrFE), we carried out XPS measurements to probe the surface composition of PEDOT:PSS, with and without the influence of paper. As noted in earlier sections, PEDOT:PSS was only found to benefit the properties of P­(VDF-TrFE) when combined with paper. Deconvoluted XPS sulfur spectra for PEDOT:PSS on glass and on paper are shown in Figure 5a. On glass, the peaks at 163.7 and 164.9 eV arise from the thiophene in PEDOT, while the peaks at 168.7and 169.9 eV are due to the sulfonic acid groups in PSS. On paper, however, the PSS peaks shift to lower binding energies, 167.9 and 169.2 eV. The peak at 165.5 eV corresponds to residual DMSO,^53^ while an additional peak at 162.5 eV on the paper substrate is thought to originate from SO_2_, which is commonly used to remove excess chlorine from bleaching.^54^ The molar content of PEDOT near the surface increases substantially, from 9.7% in PEDOT:PSS on glass to 41.2% in PEDOT:PSS on paper. This PEDOT amount is significantly higher than the amount present in the starting material (14.3%). A similar shift to lower binding energies for the PSS peaks was previously observed in PEDOT:PSS doped with cellulose nanofibers.^55^ The binding energy shift was attributed to hydrogen bonding between sulfonate groups in PSS and hydroxyl groups in cellulose. The prevalence of PEDOT at the top surface and hydrogen bonding between PSS and paper at the buried interface could indicate PSS accumulation at the interface with paper as a result of preferential bonding interactions.

**Figure 5:**
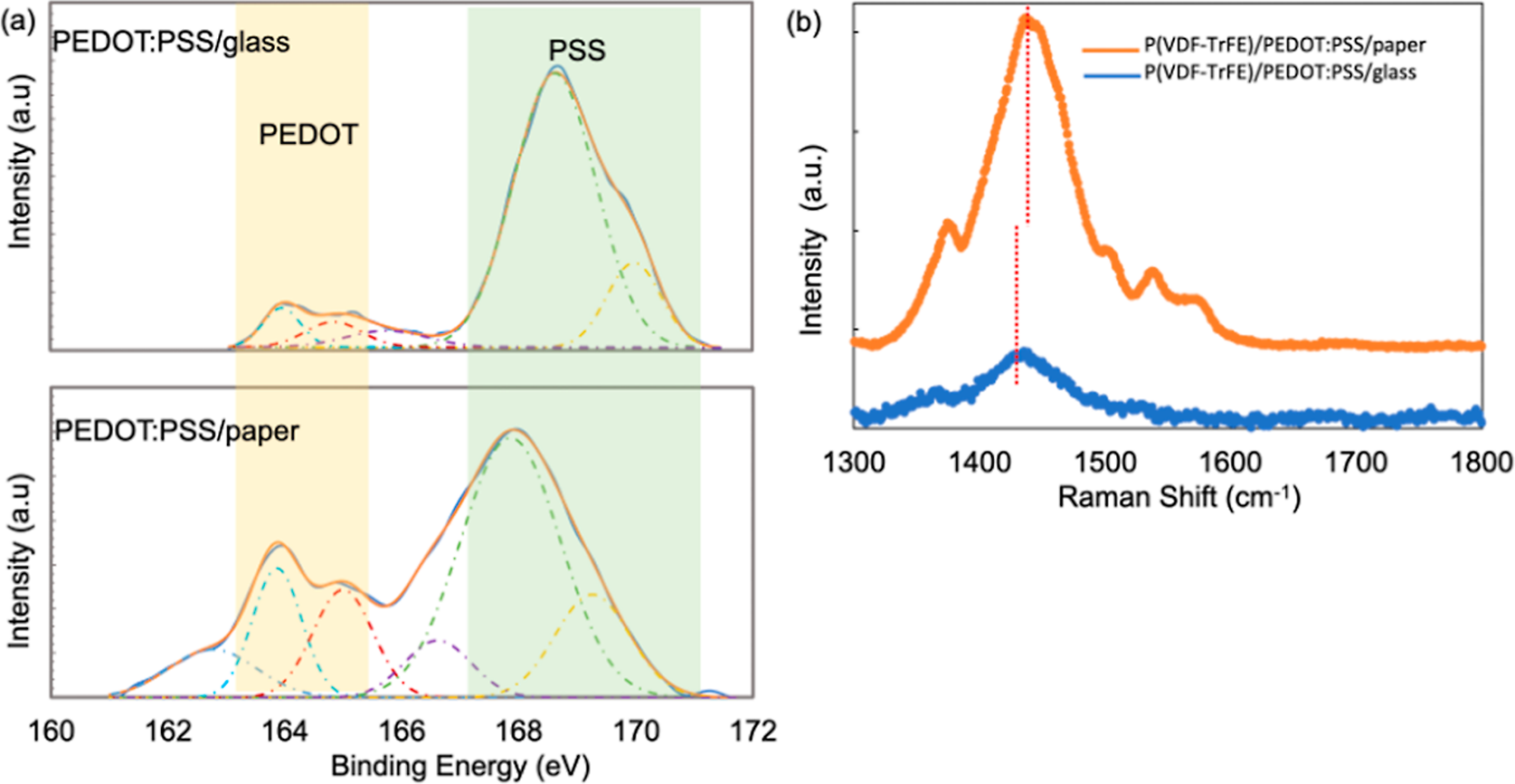
XPS sulfur spectra for (a) PEDOT:PSS/glass and PEDOT:PSS/paper. (b) Raman spectra for P­(VDF-TrFE) on PEDOT:PSS/glass and on PEDOT:PSS/paper.

The increased prevalence of PEDOT at the top surface of PEDOT:PSS/paper suggests that the PEDOT component of the PEDOT:PSS plays a central role in templating the growth of P­(VDF-TrFE). For insight into the polymer chain configuration of the PEDOT, we used Raman spectroscopy. Figure 5b shows Raman spectra for P­(VDF-TrFE) on PEDOT:PSS/paper and on PEDOT:PSS-coated glass. The peak associated with C=C bond stretching in PEDOT^56^ is at 1444 cm^–1^ for P­(VDF-TrFE) on PEDOT:PSS-coated glass and at 1434 cm^–1^ for P­(VDF-TrFE) on PEDOT:PSS/paper. These peak positions indicate that the PEDOT has a more quinoidal (linear chain) character on PEDOT:PSS/paper, while the PEDOT is in more of a benzoid (coiled) state on glass.^56^

A quinoid PEDOT structure was similarly observed in aerogels comprised of PEDOT:PSS and cellulose nanofibrils and was proposed to result from hydrogen bonding between protonated surface carboxyl groups associated with the cellulose and SO_3_H groups in PSS.^57^ Other studies have noted that the protonation of carboxyl groups in acidic PEDOT:PSS solutions also reduced the electrostatic repulsion between negatively charged cellulose and PSS, allowing van der Waals, dipole, and π–π interactions to contribute.^58,59^ By studying the temperature-dependence of PEDOT:PSS adsorption on model cellulose surfaces, a recent study suggested that adsorption is entropically driven.^60^ Collectively, these studies reflect the multifactorial nature of interactions between polyelectrolytes on cellulose and the overall consensus that interactions between PEDOT:PSS and cellulose can promote PEDOT order. On the other hand, little is known about the interactions between PEDOT:PSS and P­(VDF-TrFE).

As shown in Supporting Information Figure S10, the XPS fluorine spectrum for P­(VDF-TrFE) on PEDOT:PSS/paper exhibits a 0.66 eV shift to lower binding energy relative to P­(VDF-TrFE) onpaper. This shift can relate to interfacial interactions between P­(VDF-TrFE) and PEDOT:PSS and/or differences in dipole orientation.^61^ To elucidate the role of linear chain PEDOT in templating the orientation and ordering of P­(VDF-TrFE), we performed first-principles modeling (DFT) and *ab initio* molecular dynamics (AIMD). As shown in Figure 6, we set up models for α and β PVDF. We considered both edge-on and face-on orientations of PEDOT since our GIWAXS data shows that the PEDOT component of PEDOT:PSS adopts a distribution of orientations on paper, with 60.3% favoring an edge-on orientation and 36.6% favoring a face-on orientation (see Supporting Information Figure S9). Energetically, the β PVDF arrangements are consistently more stable compared to the α PVDF systems (Table 1). The relative energies for edge-on and face-on PEDOT are similar for β PVDF (7 meV), whereas a difference of 24 meV is seen for α PVDF.

**Figure 6:**
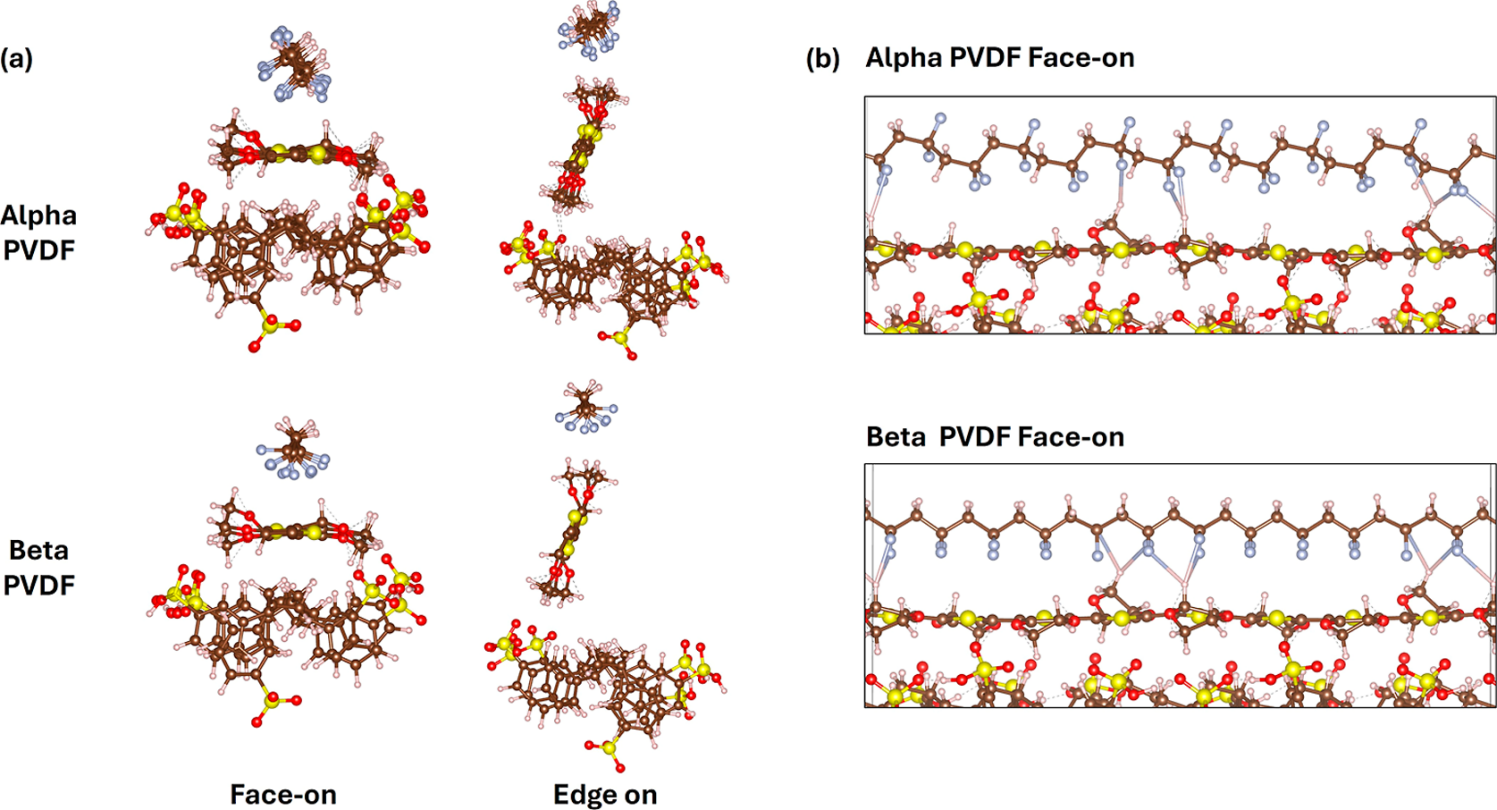
(a) Fully relaxed structures for the α and β PVDF with face-on and edge-on configurations of the PEDOT in PEDOT:PSS. (b) H-bonding with F species within a cutoff of 3.0 Å.

**Table 1:** Calculated Interaction Energies for α and β PVDF with PEDOT:PSS Normalized to the Number of Repeat Units in the PVDF Chain^a^

	interaction energy (IE)	repeat units	IE per unit	relative IE
α PVDF—face-on	–1.532	13	–0.118	0.052
α PVDF—edge-on	–1.218	13	–0.094	0.076
β PVDF—face-on	–2.042	12	–0.170	0.000
β PVDF—edge-on	–0.980	6	–0.163	0.007

a Energy values in eV. The relative energy was calculated with respect to the most stable system (β PVDF—face-on).

The formation of hydrogen bonds with the fluorine species in PVDF is more facile in the β PVDF phase due to its ability to directly align with the PEDOT chains, whereas the conformation of α PVDF leads to a more complicated structuring of the PVDF–PEDOT interface (Figure 6b). In our AIMD trajectories, we observe that the PVDF chains for both the α and β PVDF phases tend to align with the hydrogen on the PEDOT chain (Figure S15). Specifically, for face-on PEDOT interacting with β PVDF, we observe that, regardless of the starting position (fluorine facing toward or away from the PEDOT), the PVDF reorients itself to interact with the hydrogen from PEDOT. This finding provided strong evidence that hydrogen bond formation between PVDF and PEDOT is favored. It should be noted that although the hydrogen in PEDOT is bonded to carbon, which is normally a poor hydrogen bonding donor due to its weak electronegativity, effective hydrogen bonding can occur when the carbon is linked to electronegative groups such as oxygen,^62^ as is the case in PEDOT.

In our models, we observe that the most energetically stable arrangements of the PVDF chain relative to the PEDOT are those that contained the most hydrogen bonds. Initially, to test the dependence of total system energy on hydrogen bond formation, we moved a β PVDF chain along a simpler PEDOT chain (with Cl^–^ counterions replacing the PSS), while fixing the *z*-axis position of a carbon atom in the PVDF backbone (to preserve the linear positioning of the PVDF chain) (Figure S13a). Following structural relaxations, a comparison of the hydrogen bond distances (Figure S13b) reveals that the most energetically stable system forms a greater number of hydrogen bonds within a 3.0 Å cutoff,^63,64^ albeit with a higher average hydrogen bond distance. In a more realistic setup with the PSS chain as the counterion (replacing Cl^–^), we then compared the number of hydrogen bonds with the fluorine in both α and β PVDF (Figures 6b and S14). The average hydrogen bond distance follows a similar trend as discussed above, where the α PVDF forms shorter (2.56 Å versus 2.63 Å) but fewer (7 versus 10) hydrogen bonds compared to β-PVDF (Figure S14b).

To compare the hydrogen bond formation energetics in α and β PVDF chains, we considered the hydrogen bond interaction between CH_4_---FCH_3_ molecules as a reference system (Figure S16a,b). By summing the bond energies associated with each bond distance, we find that the β PVDF system shows a stronger (more negative) total hydrogen bond interaction energy than α PVDF (−0.217 eV versus −0.160 eV), supporting the conclusion that, for these systems, numerous longer hydrogen bonds win out over the case of fewer short hydrogen bonds. Consistent with the calculated interaction energy of the hydrogen bonds at play, electron-density analysis (Figure S17) shows that β PVDF chains adopt a more directional hydrogen bonding geometry with hydrogen and fluorine sites, whereas α PVDF does not, mirroring the more complicated structuring seen in α PVDF chains.

Collectively, our experiments and modeling show that the templated growth of P­(VDF-TrFE) is driven by synergistic interactions between the organic layers. The XPS and Raman data support the notion that hydrogen bonding participates in anchoring the PSS component of PEDOT:PSS to the cellulose and induces linear chain ordering and high content of PEDOT at the top PEDOT:PSS surface. GIWAXS and modeling show that the PEDOT in turn promotes β phase ordering in P­(VDF-TrFE) through further hydrogen bonding. The β phase is favored over the α phase because of the greater number of hydrogen bonding sites involved between the polymer chains.

On the other hand, when PEDOT is less abundant at the surface and not linearly ordered, templating effects were not observed. Similarly, when P­(VDF-TrFE) is deposited directly onto paper, hydrogen bonding can potentially occur between the fluorine in P­(VDF-TrFE) and the hydroxyl groups in cellulose; nevertheless, a reduced piezoelectric coefficient, β-phase content, and crystallinity were found. These observations reinforce the notion that hydrogen bonding between the P­(VDF-TrFE) and the underlying substrate is not a sufficient condition for the observed templating. Instead, we hypothesize that the commensurate relationship between the monomers in P­(VDF-TrFE) and linearly arranged PEDOT allows the PEDOT to serve as an ordered scaffold for P­(VDF-TrFE) growth. The polymer repeat distance in β-phase P­(VDF-TrFE) is 2.56–2.67 Å,^65^ while the repeat distance along linear PEDOT chains is 7.74–7.87 Å,^66^ about three times larger. As shown in our modeling data, this integer relationship between the repeat distances allows for periodic interlocking hydrogen bonds between the polymer chains.

## Conclusions

In this study, we have developed a paper-based piezoelectric composite that exhibits enhanced piezoelectric properties without electrical poling, resulting from synergistic interactions between the organic components. In the piezoelectric composite, the combined influence of wood fiber paper and the conducting polymer PEDOT:PSS lead to the templated growth of the piezoelectric polymer P­(VDF-TrFE). The out-of-plane piezoelectric coefficient of the templated P­(VDF-TrFE) is 63% higher than the untemplated case (e.g., on ITO) and 32% higher than on paper alone. The increase in piezoelectric performance in templated P­(VDF-TrFE) is associated with a sharp rise in the population of edge-on β phase crystallites, which is 5.8 times higher for P­(VDF-TrFE) on PEDOT:PSS/paper than for P­(VDF-TrFE) on paper.

The synergistic templating effect relies on PEDOT:PSS serving as a bridge between the paper and P­(VDF-TrFE). Hydrogen bonding and other supramolecular interactions between PSS and paper promote linear chain order in PEDOT, while interactions between PEDOT and P­(VDF-TrFE) promote β phase P­(VDF-TrFE) crystallization. First-principles and molecular dynamics modeling confirm the formation of hydrogen bonds between PEDOT and PVDF, with β phase PVDF being more energetically favorable than the α phase due to a greater number of available hydrogen bonds. The commensurate relationship between the polymer chains results in the PEDOT acting as a periodic hydrogen bonding scaffold for P­(VDF-TrFE) growth. The underlying paper in combination with PEDOT:PSS thus serves not only as a flexible and biocompatible substrate, but also as a macromolecular template for anchoring ordered piezoelectric polymer growth.

## Supplementary Material



## Notes

Additional AFM, PFM, FTIR, GIWAXS, XPS, DFT, and AIMD data (PDF)
