# Supplementary material for: Enhanced Piezoelectric Effect in P(VDF-TrFE) through Synergistic Templating by PEDOT:PSS and Paper
Source: ACS Appl Electron Mater. 2026 Mar 30;8(7):3153–62. doi: 10.1021/acsaelm.6c00088 (PMC13085520; doi:10.1021/acsaelm.6c00088)
Supplement: Supplementary file 1 [file el6c00088_si_001.pdf]

Supporting Information

Enhanced Piezoelectric Effect in P(VDF-TrFE) through Synergistic Templating by PEDOT:PSS and Paper

Xiangyi Wu,[a] M. D. Hashan C. Peiris,[a] Pravini S. Fernando,[b] Anju Sharma,[d] Joab Dorsainvil,[c] Ahyeon Koh,[c] Manuel Smeu,[a,b] Jeffrey M. Mativetsky*[a,b]

[a] Materials Science and Engineering, Binghamton University, Binghamton, NY, 13902, USA
[b] Department of Physics, Applied Physics and Astronomy, Binghamton University, Binghamton, NY, 13902, USA
[c] Department of Biomedical Engineering, Binghamton University, Binghamton, NY, 13902, USA
[d] Small Scale Systems Integration and Packaging Center, Binghamton University, Binghamton, NY 13902, USA

* email: jmativet@binghamton.edu

1. AFM and PFM 2
2. FTIR 5
3. GIWAXS 6
4. XPS 7
5. RDF 8
6. PSS Configuration 9
7. Chain Alignment 9
8. PVDF Positioning 10
9. H-Bond Formation 11
10. Molecular Dynamics Modeling of β PVDF-PEDOT:PSS Systems 12
11. Hydrogen Bond Interaction Energy 13
12. Spatial Electronic Density of Formation 14

1. AFM and PFM

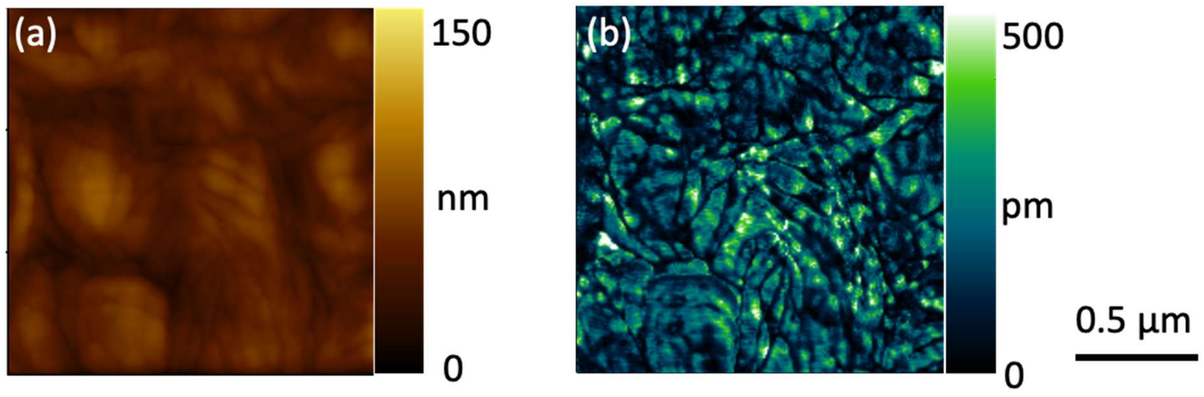

Figure S1. (a) AFM, (b) PFM measurements of P(VDF-TrFE)/PEDOT:PSS/ITO/glass.

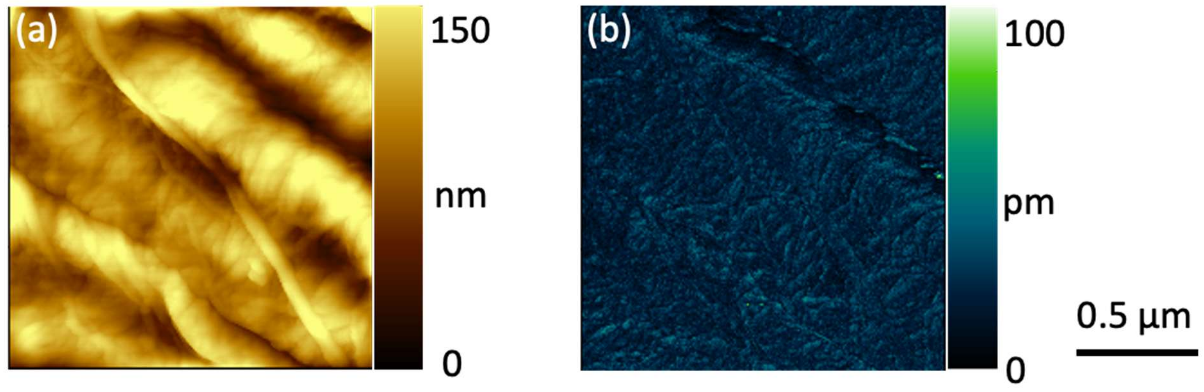

Figure S2. (a) AFM, (b) PFM measurements of pristine paper.

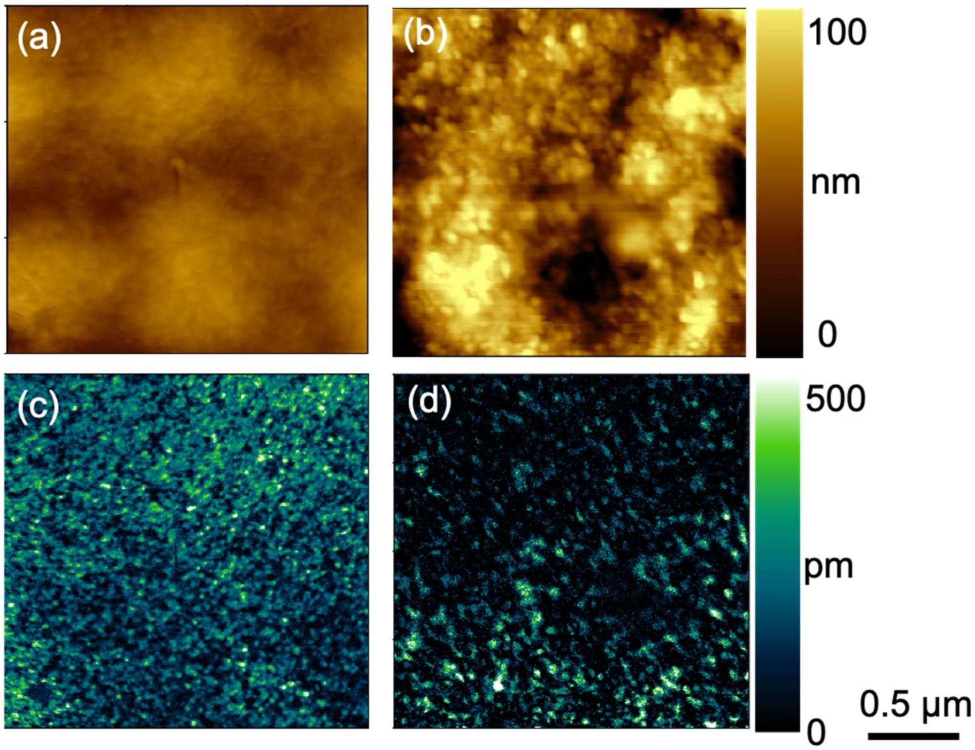

Figure S3. (a,b) AFM topography and (c,d) PFM map of P(VDF-TrFE) on PEDOT:PSS/paper dip coated using a P(VDF-TrFE) concentration of (a, c) 20 mg/ml and (b, d) 182 mg/ml. In both cases a 3.2 cm/min withdrawal speed was employed. At the lower concentration, the RMS roughness is 8.7 nm and the piezoelectric coefficient d_{33} is 39.5 pm/V, while at the higher concentration, the RMS roughness is 24 nm and d_{33} is 33.0 pm/V.

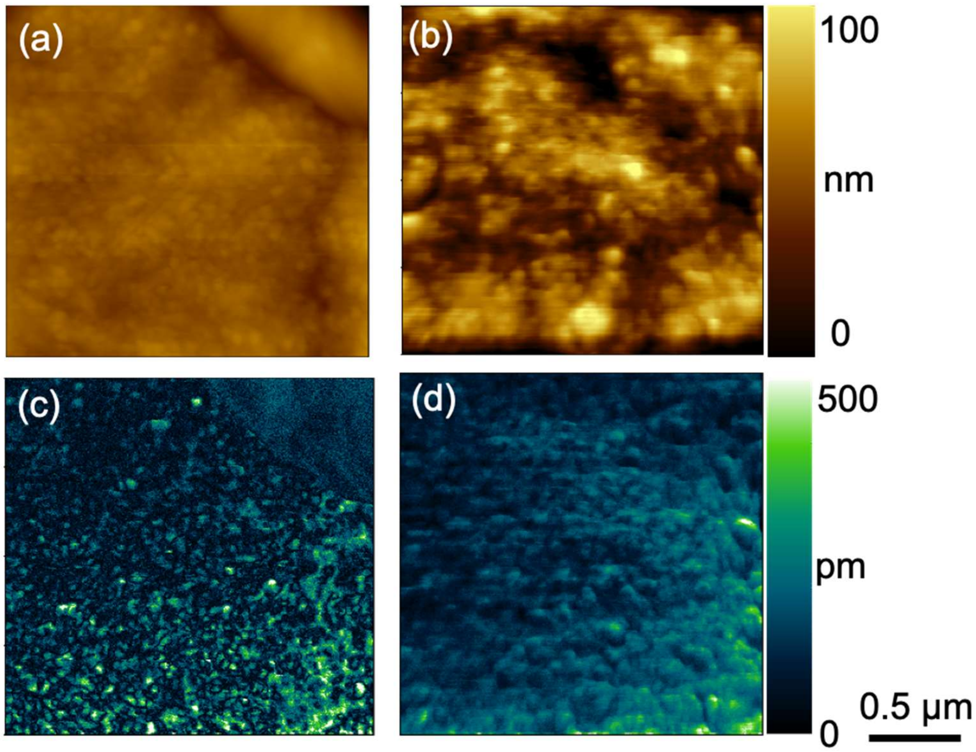

Figure S4. (a,b) AFM topography and (c,d) PFM map of P(VDF-TrFE) on PEDOT:PSS/paper dip coated using a sample withdrawal speed of (a, c) 1.0 cm/min and (b, d) 9.6 cm/min. In both cases, a P(VDF-TrFE) concentration of 62 mg/ml was employed. At the slower withdrawal speed, the RMS roughness is 9.8 nm and the piezoelectric coefficient d_{33} is 25.2 pm/V, while at the faster withdrawal speed, the RMS roughness is 20.2 nm and d_{33} is 30.4 pm/V.

2. FTIR

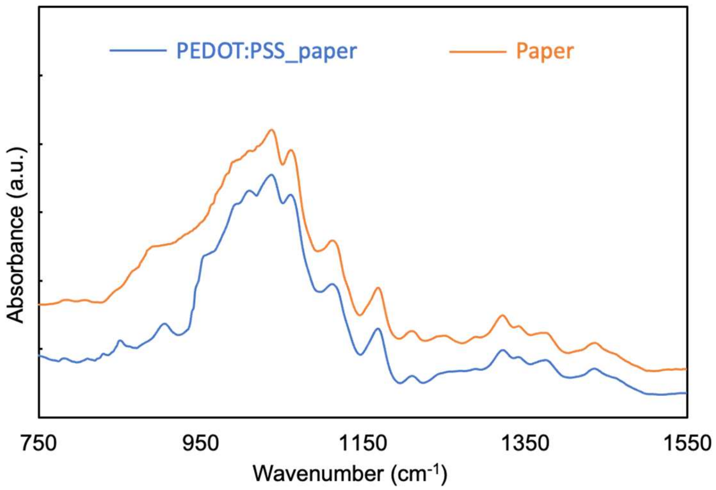

Figure S5. FTIR spectra for paper and PEDOT:PSS/paper.

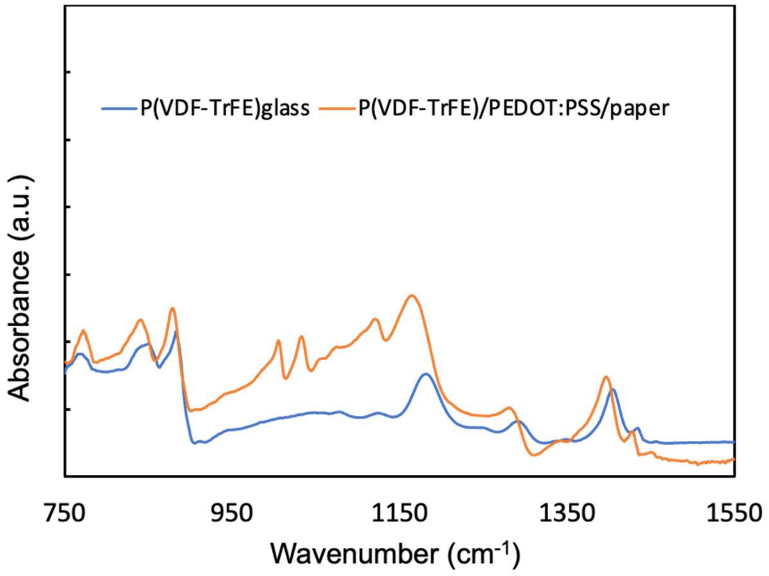

Figure S6. FTIR spectra for (a) glass and P(VDF-TrFE)/glass with the spectrum for glass subtracted; (b) PEDOT:PSS/glass and P(VDF-TrFE)/PEDOT:PSS/glass with spectrum for PEDOT:PSS/glass subtracted.

3. GIWAXS

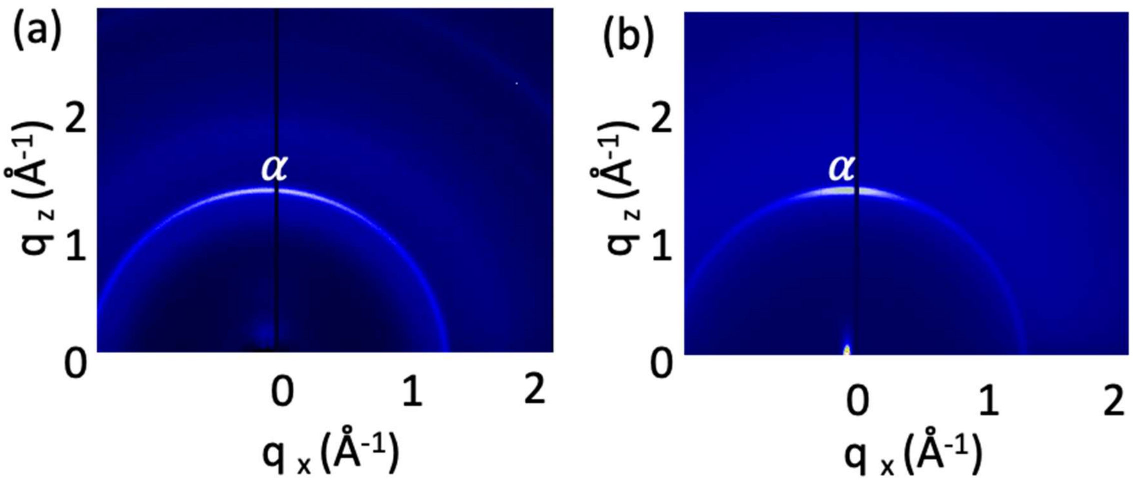

Figure S7. GIWAXS data for (a) P(VDF-TrFE)/PEDOT:PSS/glass; (b) P(VDF-TrFE)/glass

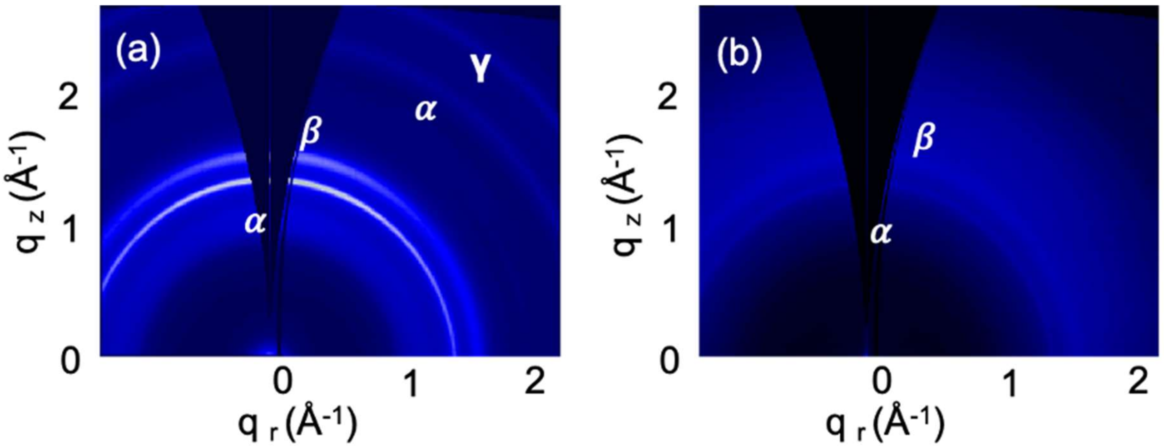

Figure S8. GIWAXS pattern corrected for the curvature of the Ewald's sphere for a (a) P(VDF-TrFE)/PEDOT:PSS/paper; and (b) P(VDF-TrFE)/paper

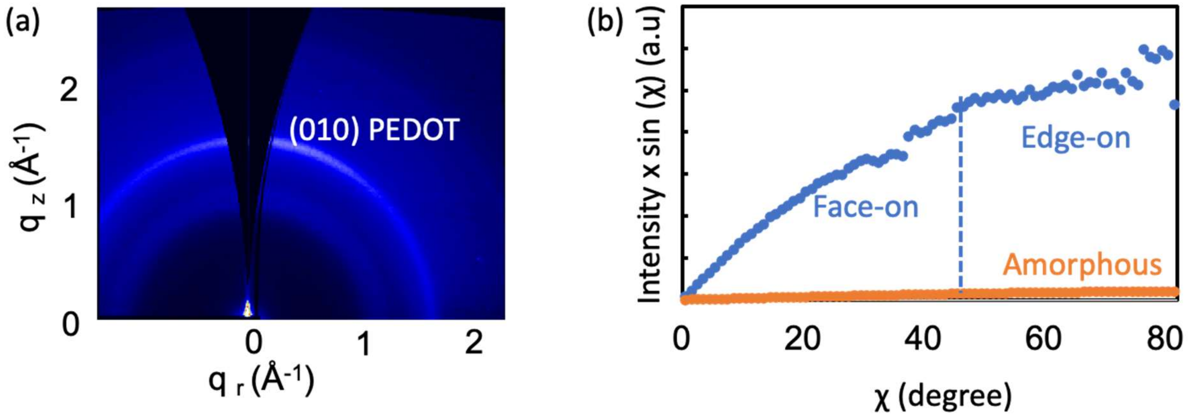

Figure S9. GIWAXS data for (a) PEDOT:PSS/paper and (b) corresponding azimuthal integration of the PEDOT (010) peak along χ, with the correction factor sin(χ).

4. XPS

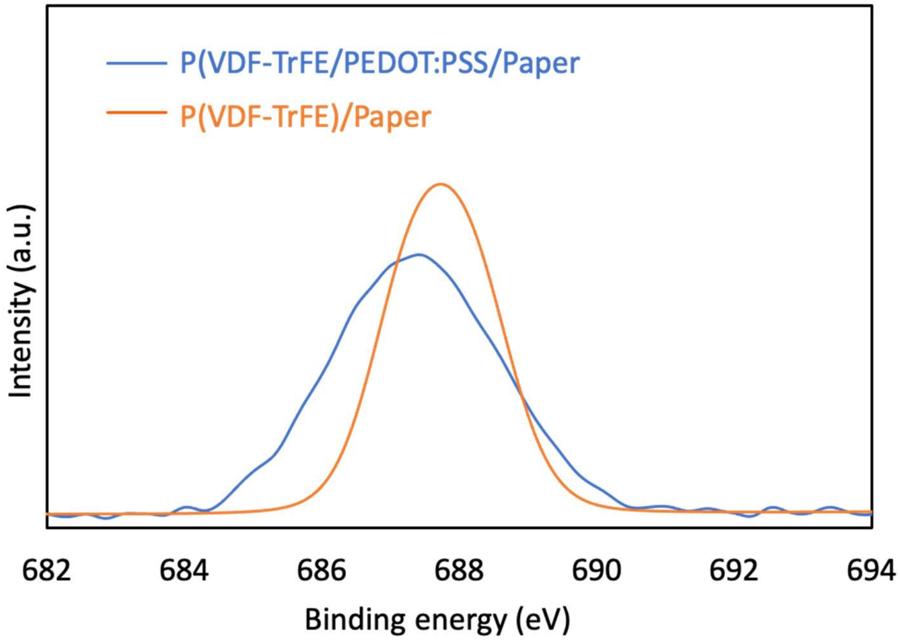

Figure S10. XPS fluorine spectra for P(VDF-TrFE)/PEDOT:PSS/paper and P(VDF-TrFE)/paper.

5. RDF

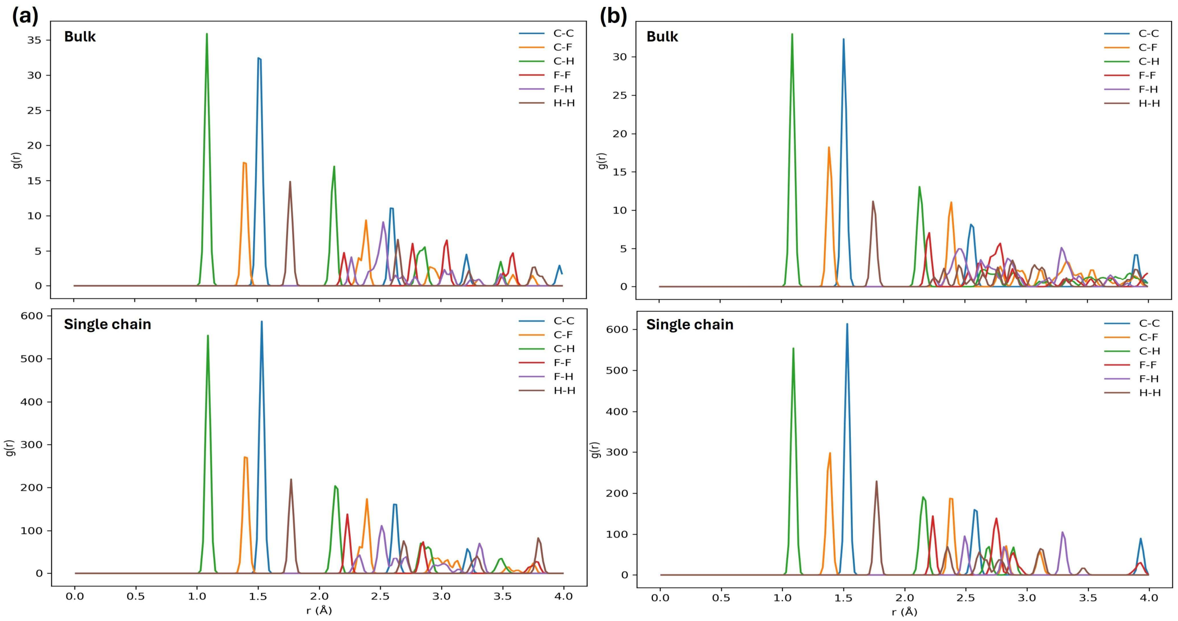

Figure S11. The radial distribution function (RDF) plots for bulk (crystalline) and single chain systems used in this study for (a) α PVDF and (b) β PVDF.

6. PSS Configuration

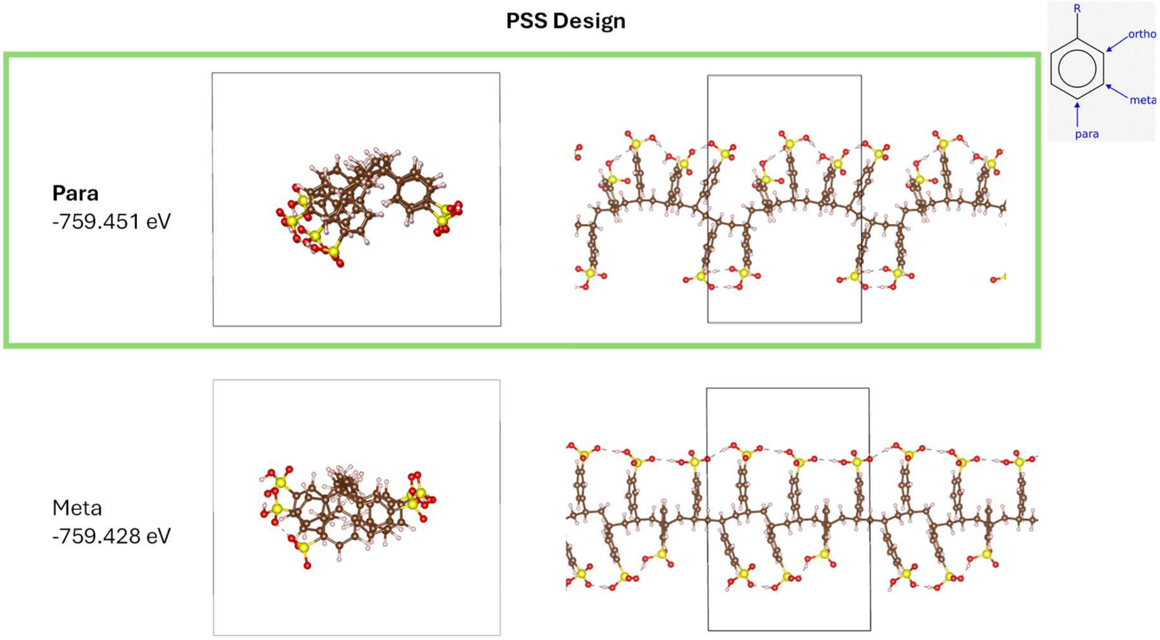

Figure S12. Fully relaxed PSS structures (charge neutral) and the comparison of the total energies for meta and para positioning of the sulfonates for the identification of the most stable positioning for the HSO_3^-. Para positioning was selected (green outline) due to it being the energetically favorable structure from our tested configurations.

7. Chain Alignment

Table S1. Lattice vector distances along the PVDF/PEDOT:PSS chains used for the calculations. Bulk refers to the fully relaxed crystalline structure.

(All distances in Å)	**Species**	**Total Length**	**Units**	**Per Unit**
**Bulk**	Alpha PVDF	13.89	6	2.32
	Beta PVDF	15.25	6	2.54
	PEDOT (Cl-)	15.34	1	15.34
	PSS	47.71	18	2.65
**Single chain**	Alpha PVDF	30.14	13	2.32
	Beta PVDF	15.36	6	2.56
	PEDOT (Cl-)	15.55	1	15.55
	PSS	14.17	6	2.36
	PSS	41.52	18	2.31

8. PVDF Positioning

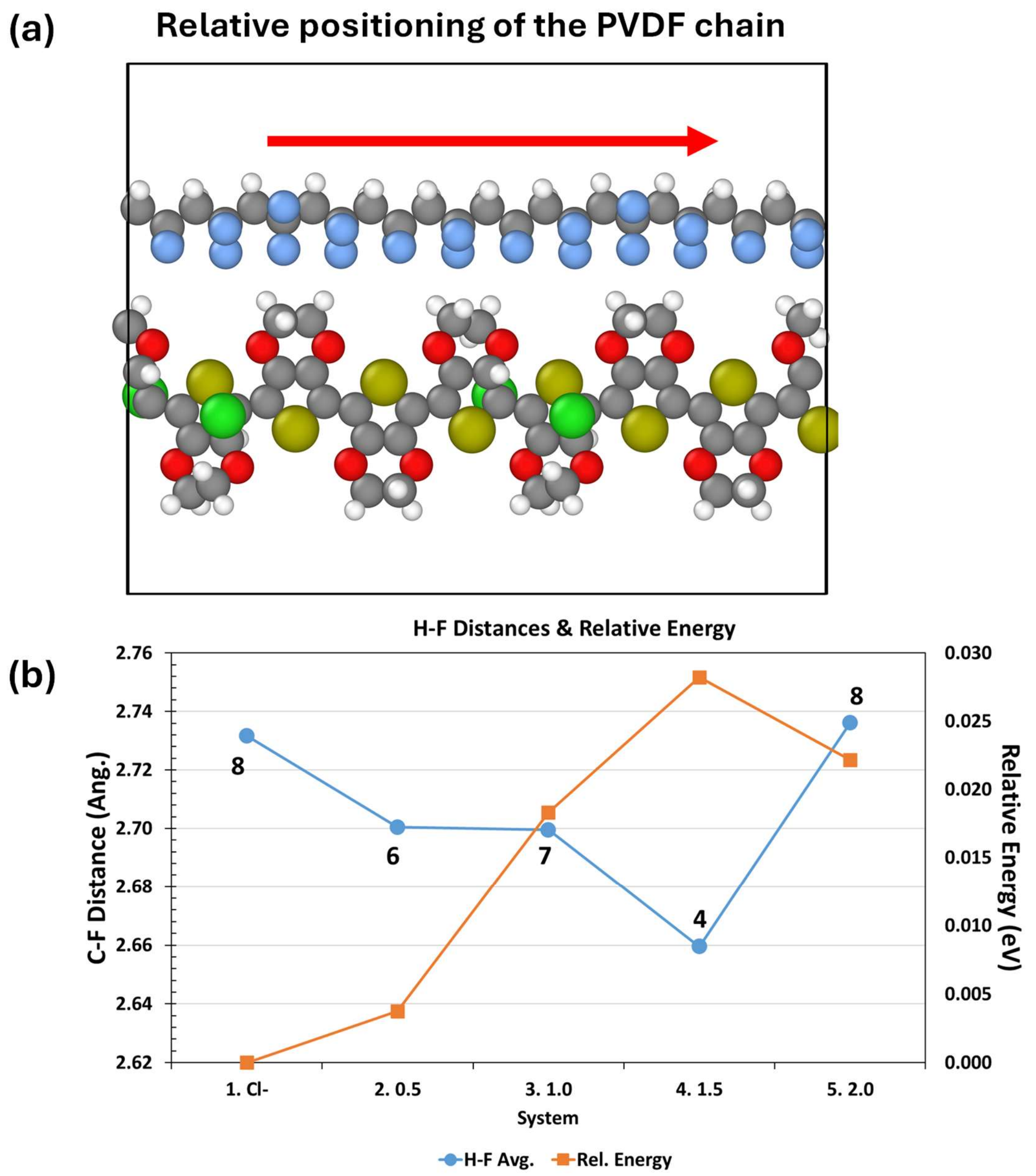

Figure S13. (a) Movement of the PVDF chain along the PEDOT chain (with Cl^- counter-ions (green)). (b) Variation of the average H-bond distance forming with the F species (3.0 Å cutoff), with the count of H—F bonds within the cutoff indicated for each positioning.

9. H-bond Formation

(a)

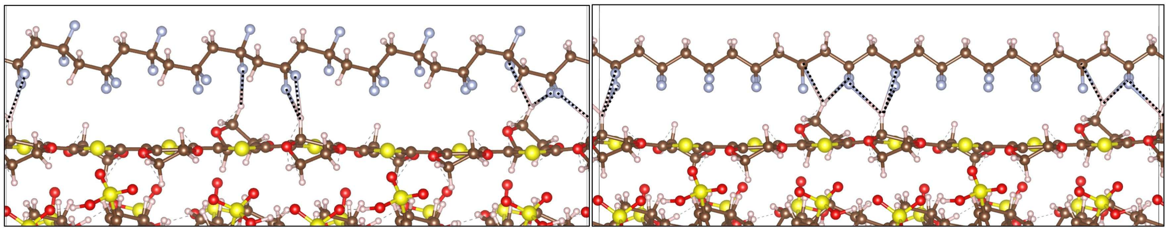

(b)

	α PVDF	β PVDF
**Avg. H-F Distance (Å)**	2.56 Å	2.63 Å
**Count**	7	10

(c)

**α PVDF**									
2.53 Å	2.58 Å	2.67 Å	2.27 Å	2.95 Å	2.71 Å	2.24 Å			
**β PVDF**									
2.58 Å	2.40 Å	2.86 Å	2.77 Å	2.51 Å	2.58 Å	2.40 Å	2.86 Å	2.77 Å	2.51 Å

Figure S14. (a) Fully relaxed structures of the α and β PVDF chains with PEDOT:PSS with H—F bonding between the chains. (b) Averaged H-bond distances for each system (3.0 Å cutoff). (c) Distances of H-bonds with the F species within the cutoff distance.

10. Molecular Dynamics Modeling of β PVDF-PEDOT:PSS Systems

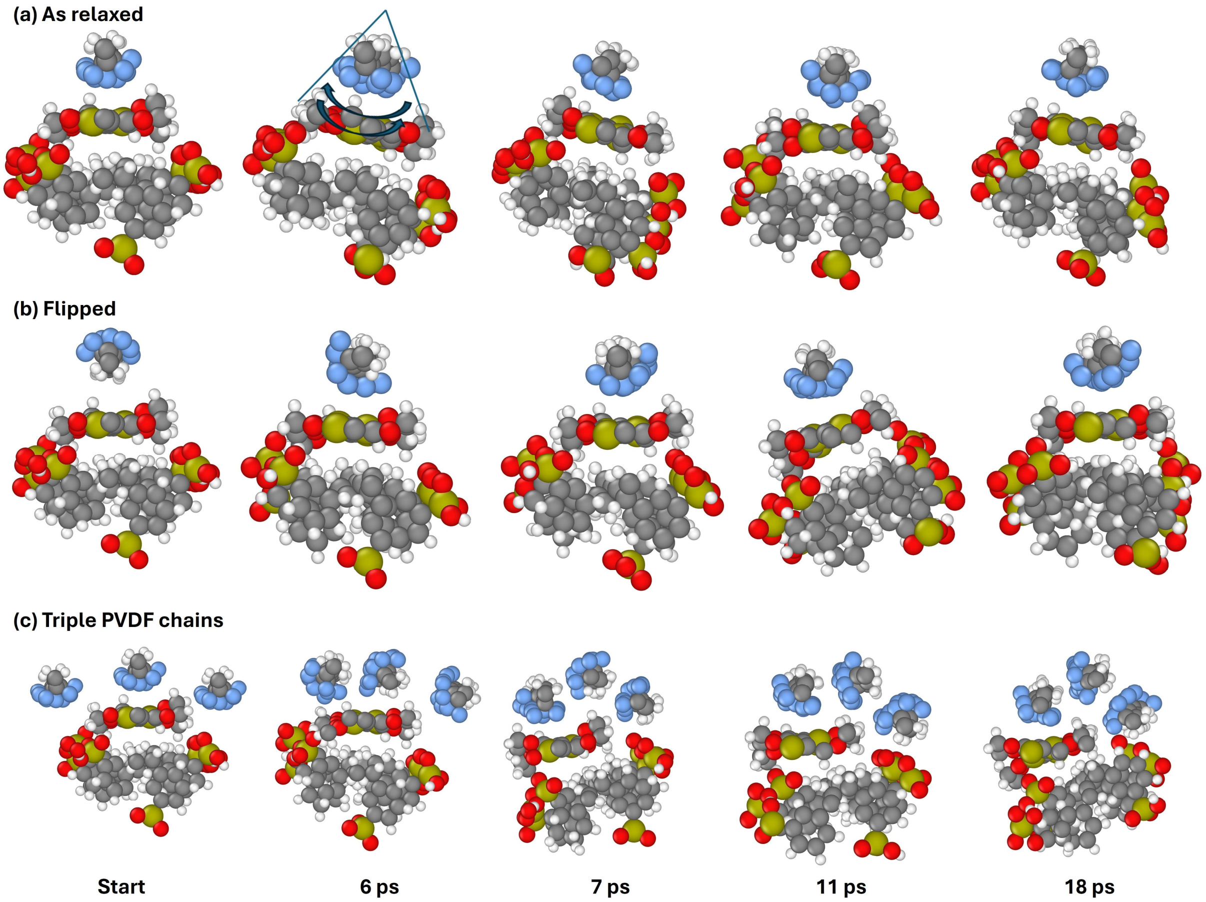

Figure S15. Dynamics of the PVDF-PEDOT:PSS chains modeled using AIMD from left to right, with the time steps listed at the bottom for (a) structure starting with the relaxed β PVDF (b) Flipped PVDF with the F species facing away from the PEDOT chain and (c) with three PVDF chains placed side by side showing how interchain H—F bonds can further stabilize the H—F interaction between PVDF and PEDOT. Note how the PVDF beginning with a flipped orientation, as shown in (b), the PVDF chains eventually realign and maintain interactions with the H of PEDOT chain.

11. Hydrogen Bond Interaction Energy

(a)

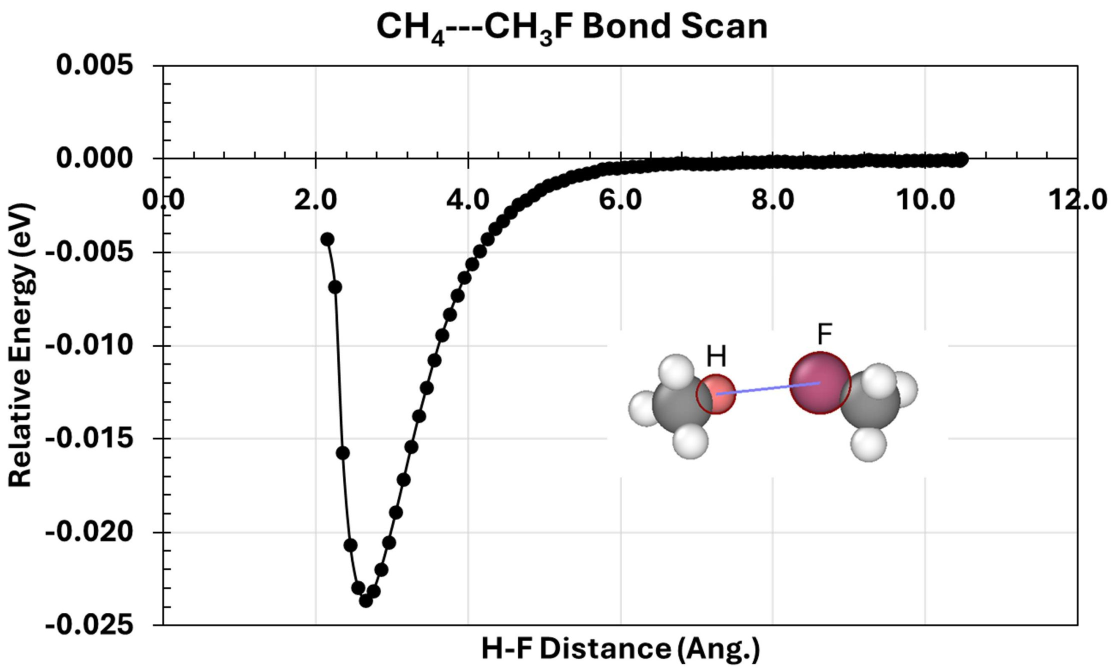

(b)

All weighted H-F Interactions - 3.5 Ang. Cutoff

	Sum of bond energies
α-PVDF	-0.160 eV
β-PVDF	-0.217 eV

Figure S16. (a) H-F interaction energy vs. bond distance for a CH_4---FCH_3 molecule pair. (b) Total of the interaction energies for H-bonds between PVDF and PEDOT within a cutoff distance of 3.5 Å.

12. Spatial Electronic Density of Formation

We calculated the volumetric charge density difference between the relaxed α and β PVDF systems (Figure S15):

$$\boldsymbol{\Delta\rho} = \boldsymbol{\rho_{composite}} - (\boldsymbol{\rho_{PVDF}} + \boldsymbol{\rho_{PSS:PEDOT}})$$

Positive isosurfaces (yellow) indicate electron accumulation and negative isosurfaces (blue) indicate electron depletion. In both systems the redistribution is localized at the contact, with electron accumulation near the F-rich PVDF surface and depletion along the PEDOT backbone and sulfonate oxygens, consistent with net charge transfer from PEDOT:PSS toward PVDF and the formation of an interfacial dipole pointing from PEDOT:PSS to PVDF. The β-PVDF case exhibits more consistent accumulation-depletion lobes than the α phase, indicating stronger interfacial polarization and therefore stronger electrostatic coupling, in line with the higher polarity of all-trans β-PVDF and with its larger interaction energy. This enhanced dipole at the β interface leads to the improvement of interfacial adhesion as calculated in Table 1 in the main article.

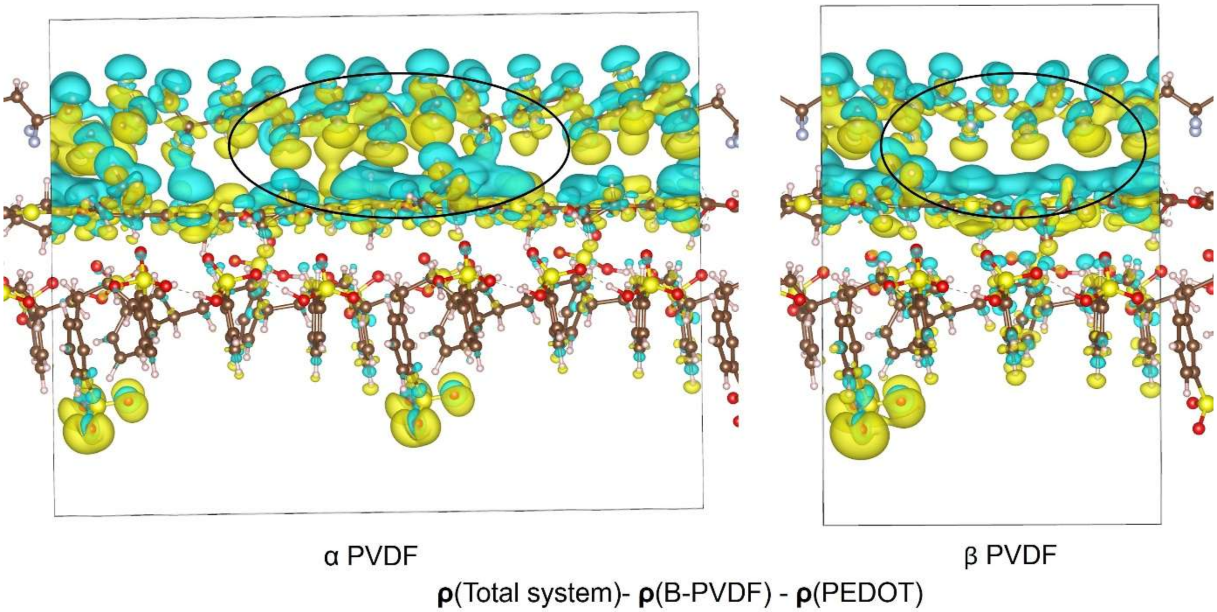

Figure S17. Charge density difference plots for the α and β PVDF systems. Yellow-positive, Blue-negative. Isosurface values were set equal for both images.
